# Calcitonin Inhibits SDCP-Induced Osteoclast Apoptosis and Increases Its Efficacy in a Rat Model of Osteoporosis

**DOI:** 10.1371/journal.pone.0040272

**Published:** 2012-07-06

**Authors:** Yi-Jie Kuo, Fon-Yih Tsuang, Jui-Sheng Sun, Chi-Hung Lin, Chia-Hsien Chen, Jia-Ying Li, Yi-Chian Huang, Wei-Yu Chen, Chin-Bin Yeh, Jia-Fwu Shyu

**Affiliations:** 1 Department of Orthopaedic, Taipei Medical University Hospital, Taipei, Taiwan, Republic of China; 2 Institute of Clinical Medicine, National Yang Ming University, Taipei, Taiwan, Republic of China; 3 Division of Neurosurgery, Department of Surgery, National Taiwan University Hospital, Taipei, Taiwan, Republic of China; 4 Department of Orthopaedic Surgery, National Taiwan University Hospital-Hsin Chu, Hsin-Chu, Taiwan, Republic of China; 5 Graduate Institute of Clinical Medicine, College of Medicine, Taipei Medical University, Taipei, Taiwan, Republic of China; 6 Institute of Microbiology and Immunology, National Yang Ming University, Taipei, Taiwan, Republic of China; 7 Department of Biology and Anatomy, National Defense Medical Center, Taipei, Taiwan, Republic of China; 8 Institute of Anatomy and Cell Biology National Yang Ming University, Taipei, Taiwan, Republic of China; 9 Department of Psychiatry, Tri-Service General Hospital, Taipei, Taiwan, Republic of China; Cinvestav-IPN, Mexico

## Abstract

**Introduction:**

Treatment for osteoporosis commonly includes the use of bisphosphonates. Serious side effects of these drugs are caused by the inhibition of bone resorption as a result of osteoclast apoptosis. Treatment using calcitonin along with bisphosphonates overcomes these side-effects in some patients. Calcitonin is known to inhibit bone resorption without reducing the number of osteoclasts and is thought to prolong osteoclast survival through the inhibition of apoptosis. Further understanding of how calcitonin inhibits apoptosis could prove useful to the development of alternative treatment regimens for osteoporosis. This study aimed to analyze the mechanism by which calcitonin influences osteoclast apoptosis induced by a bisphosphate analog, sintered dicalcium pyrophosphate (SDCP), and to determine the effects of co-treatment with calcitonin and SDCP on apoptotic signaling in osteoclasts.

**Methods:**

Isolated osteoclasts were treated with CT, SDCP or both for 48 h. Osteoclast apoptosis assays, pit formation assays, and tartrate-resistant acid phosphatase (TRAP) staining were performed. Using an osteoporosis rat model, ovariectomized (OVX) rats received calcitonin, SDCP, or calcitonin + SDCP. The microarchitecture of the fifth lumbar trabecular bone was investigated, and histomorphometric and biochemical analyses were performed.

**Results:**

Calcitonin inhibited SDCP-induced apoptosis in primary osteoclast cultures, increased Bcl-2 and Erk activity, and decreased Mcl-1 activity. Calcitonin prevented decreased osteoclast survival but not resorption induced by SDCP. Histomorphometric analysis of the tibia revealed increased bone formation, and microcomputed tomography of the fifth lumbar vertebrate showed an additive effect of calcitonin and SDCP on bone volume. Finally, analysis of the serum bone markers CTX-I and P1NP suggests that the increased bone volume induced by co-treatment with calcitonin and SDCP may be due to decreased bone resorption and increased bone formation.

**Conclusions:**

Calcitonin reduces SDCP-induced osteoclast apoptosis and increases its efficacy in an in vivo model of osteoporosis.

## Introduction

Bisphosphonates are the most commonly prescribed first line medication for osteoporosis despite causing side effects, including low bone turnover, hypocalcemia, and osteonecrosis of the jaw due to decreased bone formation as well as increased bone fracture due to reduced bone resorption [1], [2]. Although the molecular mechanisms by which they inhibit bone resorption vary among the bisphosphonates, they collectively induce osteoclast apoptosis. Specifically, simple bisphosphonates are incorporated into non-hydrolysable adenosine triphosphate analogues, inducing osteoclast apoptosis [3]. The more potent nitrogen-containing bisphosphonates inhibit farnesyl pyrophosphate synthase, a key enzyme of the mevalonate pathway, which is essential for protein prenylation in osteoclasts [3], [4]. Thus, bisphosphonates inhibit bone resorption by disrupting osteoclast function and survival.

Calcitonin has also been used as a therapy for osteoporosis, hypercalcemia, and Paget’s disease. This 32-amino-acid peptide hormone induces hypocalcemia by inhibiting osteoclast-induced bone resorption. Although it has been used for nearly 30 years, it is less widely used than bisphosphonates and estrogen [5]–[7]. In addition, the physiological role of calcitonin in calcium homeostasis and bone remodeling as well as its effects on bone cells remains unclear. For example, studies using calcitonin-null mice indicate that it may be involved in protecting the skeleton during periods of “calcium stress”, such as growth, pregnancy, and lactation [8]. However, in the basal state, only modest effects on regulating bone remodeling and calcium homeostasis were observed [9]. Furthermore, calcitonin primarily inhibits bone resorption [10], [11] without reducing the number of osteoclasts [12]. Although the apoptotic signaling pathways regulated by calcitonin in osteoclasts remain to be fully elucidated, the phosphokinase A (PKA) pathway is likely involved [13]. In addition, calcitonin protects osteoclasts from the effects of a nitric oxide-releasing compound, a highly effective apoptotic stimulus [14]. Downregulation of Cox activity by calcitonin inhibits the function, but not survival of osteoclasts [15]. However, it may also interfere with bone remodeling by inhibiting bone formation [16], [17] although not markedly in humans [1]. Combined use of calcitonin and anti-resorptive agents with different modes of action may overcome the side-effects experienced by some patients taking bisphosphonates.

Sintered dicalcium pyrophosphate (SDCP) is a pyrophosphate analog developed by Lin et al. [18]. It was proven biocompatible with bone in an in vivo animal model [18] and in vitro cell culture model [19]. Furthermore, in ovariectomized rats, SDCP increased bone mass [20] by inducing osteoclast apoptosis [21]. Moreover, the effects of SDCP were comparable to those observed for alendronate, a bisphosphonate commonly used clinically [20]. However, further studies are necessary to fully elucidate its mechanism of action.

Because calcitonin may prolong osteoclast survival through inhibition of apoptosis, this study aimed to analyze its influence on osteoclast apoptosis induced by a bisphosphate analog, SDCP. Specifically, the effects of calcitonin and SDCP co-treatment on osteoclast apoptosis and survival were assessed. In addition, the mechanism by which calcitonin influences SDCP-induced apoptosis of osteoclasts was determined. Finally, this study aimed to investigate the potential synergistic effects of calcitonin and SDCP co-treatment in ovariectomized rats by assessing bone volume, trabecular number, thickness, and separation as well as bone formation. Because the present osteoporosis treatments, including bisphosphonates, have side effects, the investigation of such potential alternative treatments is warranted. The examination of the effects of co-treatment with calcitonin and SDCP on osteoclast apoptotic signaling may help elucidate the mechanism by which calcitonin exerts its antiapoptotic effect in osteoclasts.

## Results

### Effects of Calcitonin and SDCP on Osteoclast Apoptosis

**Figure 1:**
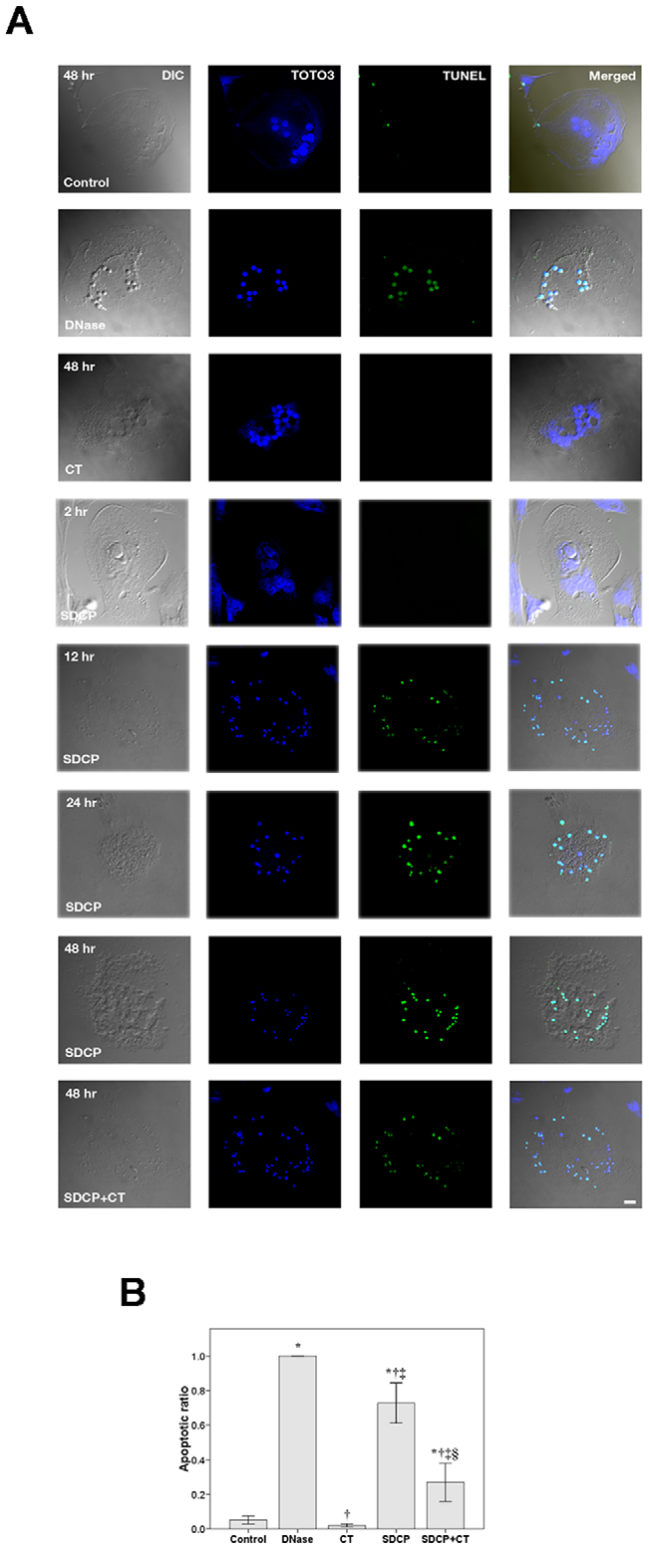
Detection of osteoclast apoptosis using TUNEL analysis. (A) Osteoclasts were treated with CT (10 nM), SDCP (10 µM), or both for the times indicated. Osteoclasts treated with DNase I (3 U/mL) were included as positive controls. Green nuclear labeling indicates apoptotic cells. Nuclei were counterstained using TOTO-3 (blue). Difference interference contrast (DIC) images show morphology of the cells. Bar = 20 µm. (B) Quantitative results of the experiment shown in panel A. *P*<0.05 compared to *control group, †DNase group, ‡CT group, and §SDCP group after Bonferroni adjustment, mean ± SD, n = 6 in each group.

As shown in Fig. 1, osteoclast apoptosis was assessed using the TUNEL assay. Confocal analysis of osteoclasts cultured in control medium for 48 h revealed approximately 5% of TUNEL stain-positive cells (Fig. 1A and B). SDCP induced a time-dependent increase in TUNEL stain-positive cells, which became apparent after 12 hours and reached a maximum level at 48 hours of treatment. Both DNase and SDCP treatment increased TUNEL stain-positive cells to 100.0±0% and 72.8±11.6%, respectively (Fig. 1B). As compared to control and SCDP-treated cells, addition of calcitonin significantly decreased the number of TUNEL-positive osteoclasts (2.0±0.9 vs. 5.2±2.3% and 27.0±11.1 vs. 72.8±11.6%, respectively; Fig. 1B).

**Figure 2:**
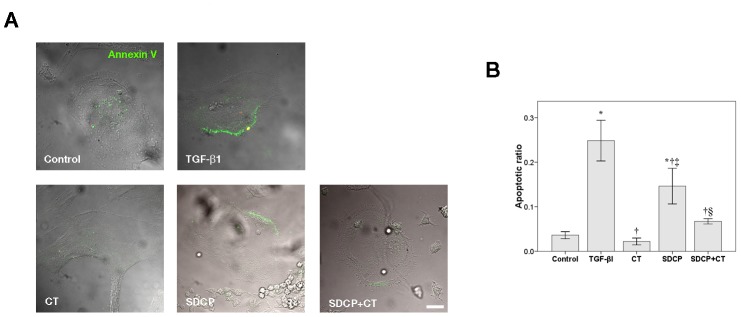
Detection of apoptosis by annexin V labeling in osteoclasts. (A) Osteoclasts were treated with CT (10 nM), SDCP (10 µM), or both for 18 h. Osteoclasts treated with TGF-βI (10 ng/mL) were included as positive controls. Green cellular membrane labeling indicates apoptotic cells. Only cells without propidium iodide (red) labeling were considered to be apoptotic. DIC images and nucleus stain were performed as described in Fig. 1. Bar = 10 µm. (B) Quantitative results of the experiment shown in panel A. The measurement of apoptosis was calculated as the percentage of positive annexin V labeling cells in a total of at least 500 osteoclasts. *P*<0.05 compared to *control group, †TGF-β1 group, ‡CT group; and §SDCP group after Bonferroni adjustment, mean ± SD, n = 6 in each group.

Apoptosis was also assessed using annexin-V staining (Fig. 2). In osteoclasts cultured for 18 h, treatment with TGF-β1, the positive control, and SDCP increased annexin-V-positive cells by 24.9±4.6% and 14.6±4.0%, respectively. Upon cotreatment with calcitonin and SDCP, a significant decrease in annexin-V-positive cells was observed as compared with those cells treated with SDCP alone (6.7±0.6 vs. 14.6±0.4%; Fig. 2A and B). Because these data demonstrate that SDCP induced osteoclast apoptosis, the TGF-β1 treatment group was eliminated from subsequent experiments.

### Calcitonin Inhibits SDCP-induced Expression of Cleaved Caspase 3 in Osteoclasts

**Figure 3:**
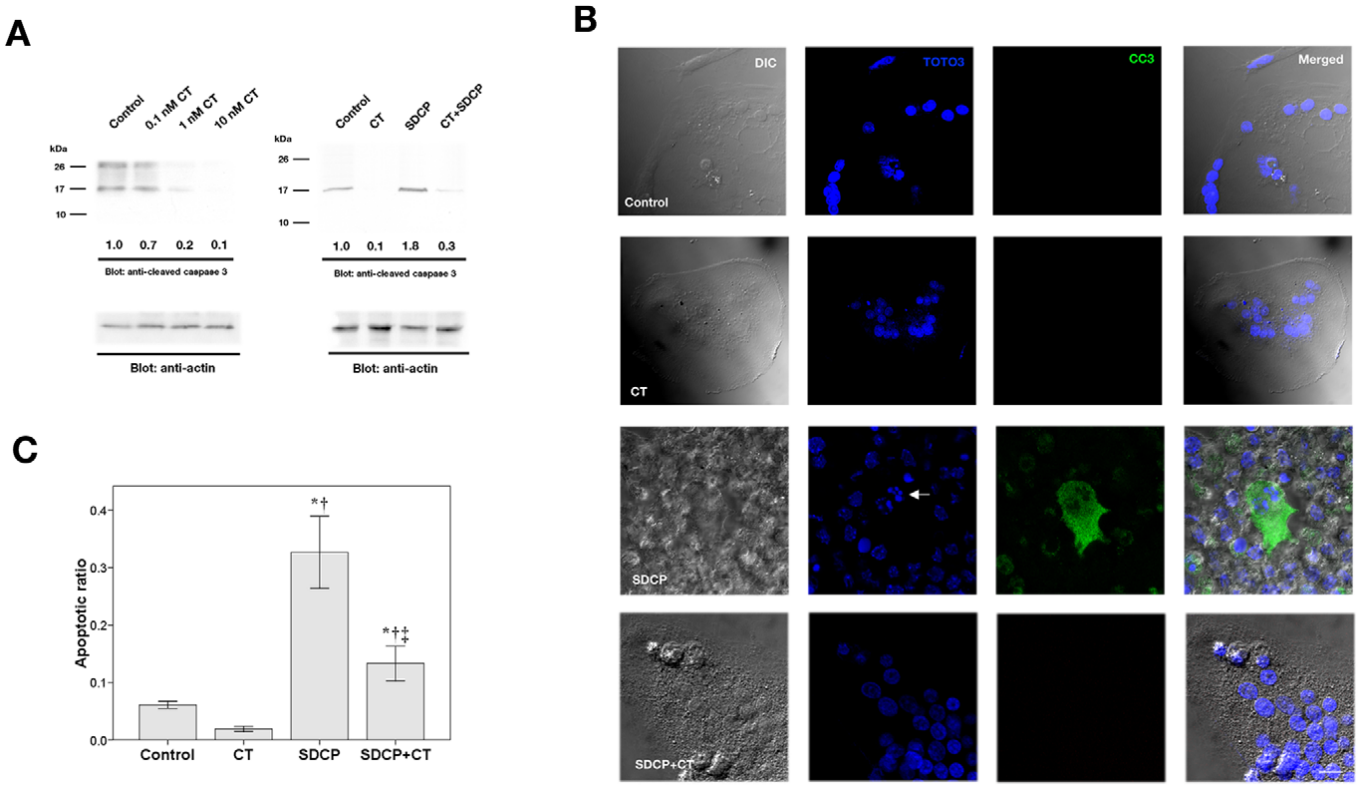
Calcitonin inhibits SDCP-induced expression of cleaved caspase 3 in osteoclasts. (A) Western blot analysis of cleaved caspase 3 expression in osteoclasts treated with CT (10 nM), SDCP (10 µM), or both. Protein levels were quantified by densitometry, corrected for the sample load based on actin expression, and expressed as fold-increase or decrease relative to the control lane. Each blot is representative of at least three replicate experiments. (B) Confocal analysis of immunofluorescent labeling of cleaved caspase 3 in calcitonin- or SDCP-treated osteoclast. Green intracellular cleaved caspase 3 labeling and blue TOTO3-labeled nuclear chromatin condensation (white arrow) indicate cells that underwent apoptosis. Bar = 10 µm. (C) Quantitative results of the experiment shown in panel B. The measurement of apoptosis was calculated as a percentage of positive cleaved caspase-3–labelled cells in a total of at least 500 osteoclasts. P<0.05 compared to *control group, † CT group, and ‡SDCP group after Bonferroni adjustment, mean ± SD, n = 6 in each group.

To determine the effects of SDCP and calcitonin on caspase-3 cleavage, Western blot analysis was employed (Fig. 3). Exposure of osteoclasts to 0.1, 1 and 10 nM calcitonin for 18 h induced a dose-dependent decrease of cleaved caspase-3 (Fig. 3A). In addition, exposure of osteoclasts to 10 µM SDCP induced an increase in cleaved caspase-3, which was inhibited with the addition of 10 nM calcitonin (Fig. 3A). Cleaved caspase-3 was also assessed using confocal analysis of immunofluorescent-labeled cells; decreased cleaved caspase-3 labeling was observed in osteoclasts treated with calcitonin and SDCP as compared to those treated with SDCP alone (13.4±1.5 vs. 32.9±3.2%; Fig. 3B and C).

### Regulation of Apoptosis-related Protein Expression and Activation by Calcitonin and SDCP

**Figure 4:**
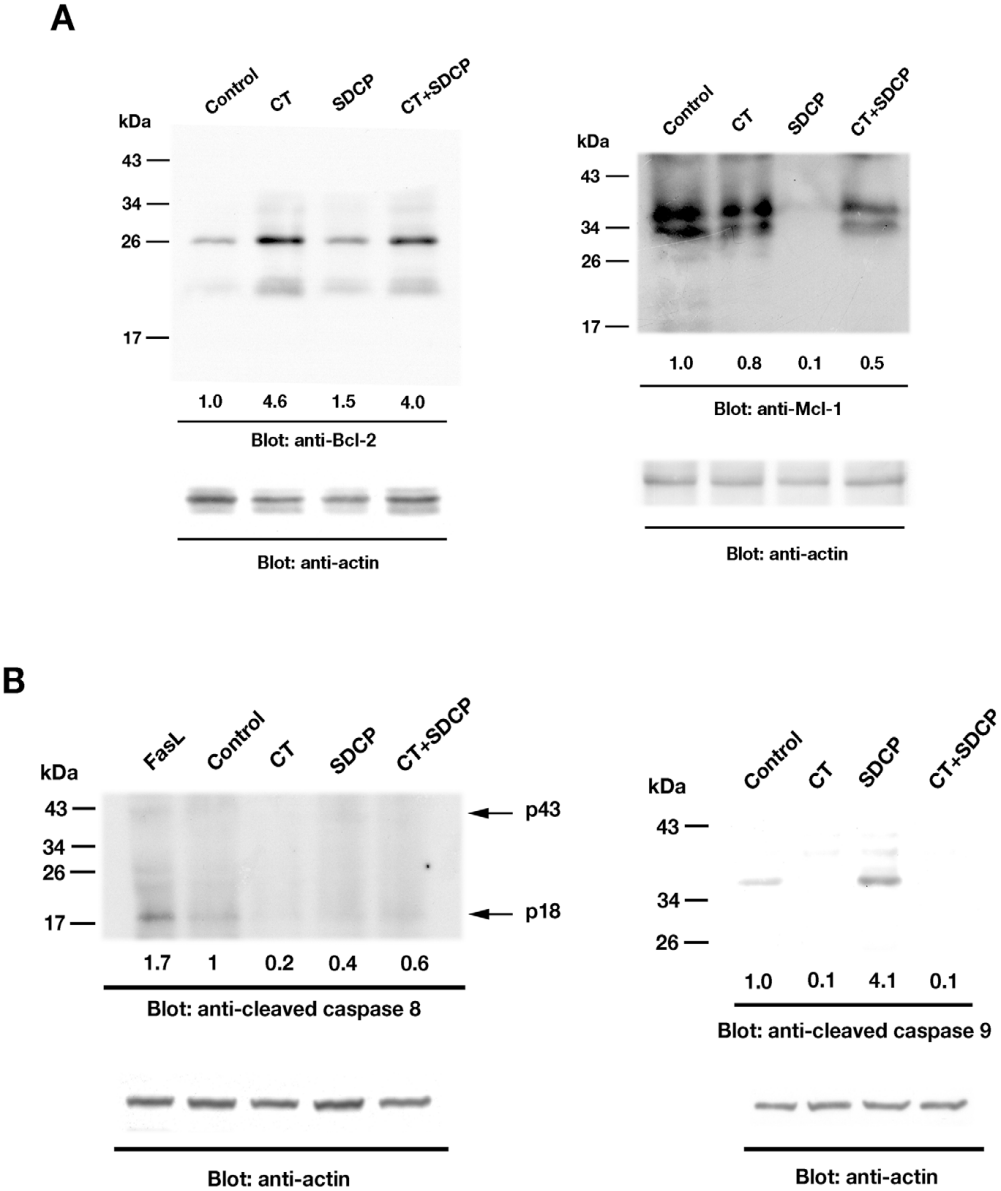
Regulation of apoptotic signaling by calcitonin and SDCP. (A) Western blot analysis of Bcl-2 and Mcl-1 expression in osteoclasts treated with CT (10 nM), SDCP (10 µM), or both. (B) Caspase 8 and 9 activation in response to CT (10 nM) or SDCP (10 µM) stimulation. Western blot analysis revealed the presence of two fragments (p43 and p18), corresponding to the cleaved caspase 8 under the treatment of FasL (10 ng/mL). Western blot analysis of cleaved caspase 9 was shown in the right panel. Protein levels were quantified by densitometry, corrected for the sample load based on actin expression, and expressed as fold-increase or decrease relative to the control lane. Each blot is representative of at least three replicate experiments.

The effects of calcitonin and SDCP on Bcl-2 and Mcl-1 expression as well as caspase cleavage were assessed using Western blot analysis (Fig. 4). Calcitonin alone and with SDCP increased Bcl-2 expression in osteoclasts; SDCP only slightly increased Bcl-2 expression (Fig. 4A). However, SDCP decreased Mcl-1 expression, which was partially reversed by the addition of calcitonin with the SDCP (Fig. 4A); calcitonin alone only slightly reduced Mcl-1 expression (Fig. 4A). FasL, the positive control, increased caspase-8 cleavage whereas both calcitonin and SDCP induced little activation of caspase-8 in osteoclasts (Fig. 4B). Calcitonin decreased and SDCP increased caspase-9 cleavage in osteoclasts, which was inhibited by calcitonin co-treatment (Fig. 4B). Because this data indicates that SDCP induces apoptosis through a pathway distinct from FasL, the FasL treatment group was eliminated from subsequent experiments.

### Calcitonin Inhibits Osteoclast Apoptosis through Bcl-2 and Erk Signaling Pathway

**Figure 5:**
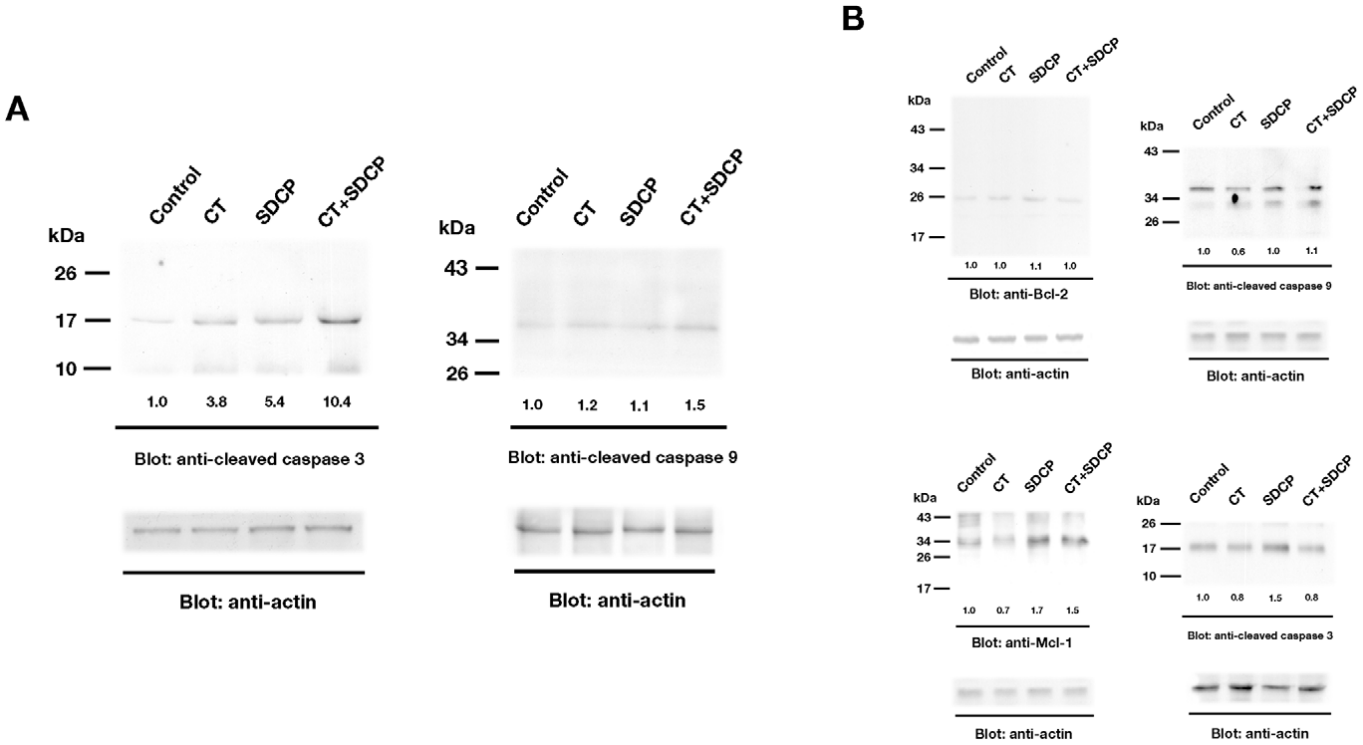
Calcitonin inhibits osteoclast apoptosis through Bcl-2 and Erk signaling. (A) Western blot analysis of expression of caspase 3 and 9. Osteoclasts were first treated with HA14-1 (50 µM) for 2 h and then CT (10 nM), SDCP (10 µM), or both were added to the culture medium. (B) Examination of apoptotic signaling regulated by calcitonin and SDCP in the presence of an Erk inhibitor. Osteoclasts were first treated with PD98059 (5 µM) for 2 h and then CT (10 nM), SDCP (10 µM), or both were added to the culture medium. Protein levels were quantified by densitometry, corrected for the sample load based on actin expression, and expressed as fold-increase or decrease relative to the control lane. Each blot is representative of at least three replicate experiments.

As shown in Fig. 5, the effects of calcitonin and SDCP treatment on apoptosis-related signaling pathways were determined by Western blot analysis. Pretreatment of osteoclasts with HA14-1, a Bcl-2 inhibitor, blocked the reduction in cleaved caspase-3 and -9 induced by calcitonin treatment (Fig. 5A). In addition, pre-treatment of osteoclasts with PD98059, an Erk1/2 inhibitor, blocked the calcitonin-induced increase of Bcl-2 and SDCP-induced decrease of Mcl-1 (Fig. 5B). The SDCP-induced increase of cleaved caspase-9 was also blocked by PD98059 pre-treatment, but the calcitonin-induced decrease was not. Finally, the decreased cleaved caspase-9 by cotreatment with calcitonin and SDCP as well as the calcitonin-induced decrease of cleaved caspase-3 were sensitive to PD98059 pretreatment (Fig. 5B).

### Calcitonin Reduces the Inhibitory Effect of SDCP on Osteoclast Survival but not Activity

**Figure 6:**
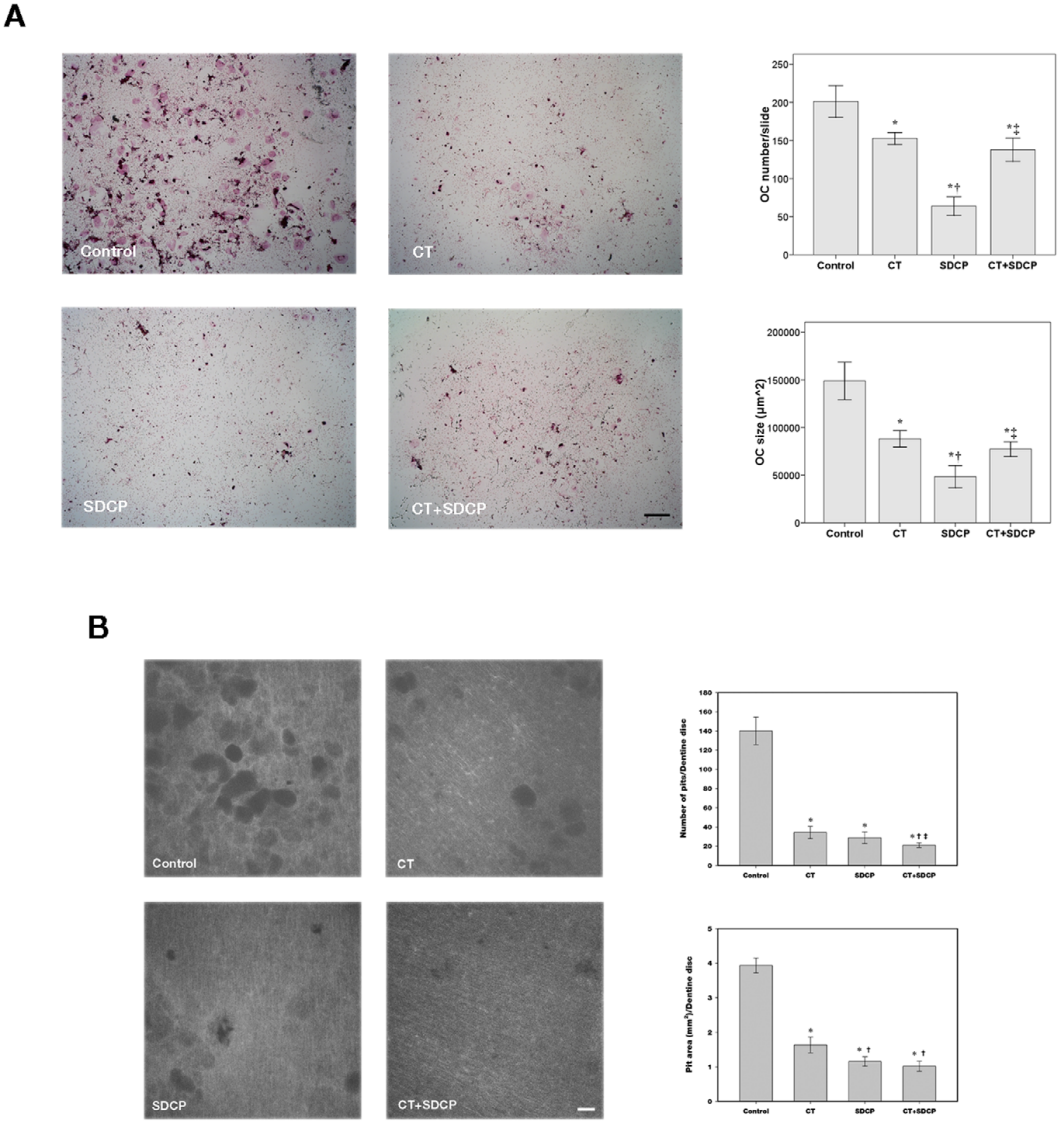
Calcitonin reduces inhibitory effect of SDCP on osteoclast survival but not activity. (A) TRAP stain of osteoclasts treated with CT (10 nM), SDCP (10 µM), or both. Red intracellular stain with multiple nuclei indicates positive labeling. Quantitative results of cell number and size were shown in right panel. Bar = 500 µm. (B) Pit formation assay. Osteoclasts were cultured on dentine discs and treated with CT (10 nM), SDCP (10 µM), or both. Quantitative results of the number and area of resorption pits were shown in right panel. Bar = 50 mm. *P*<0.05 compared to *control group, † CT group, and ‡SDCP group after Bonferroni adjustment, mean ± SD, n = 6 in each group.

The effects of calcitonin and SDCP cotreatment on osteoclast number, size and bone resorption were determined (Fig. 6). In osteoclasts treated with calcitonin, TRAP staining revealed a significant decrease in cell number and size as compared to those cells in control medium, which was further decreased with SDCP treatment alone. However, combined treatment of calcitonin and SDCP alleviated the SDCP-induced decrease in osteoclast number and size (Fig. 6A). A similar inhibitory effect on pit number and area was observed on dentine discs in the calcitonin and SDCP alone groups. Combined treatment of calcitonin and SDCP induced a further decrease in pit number (Fig. 6B).

### Calcitonin Increases the Therapeutic Efficacy of SDCP in an in vivo Osteoporosis Model

**Figure 7:**
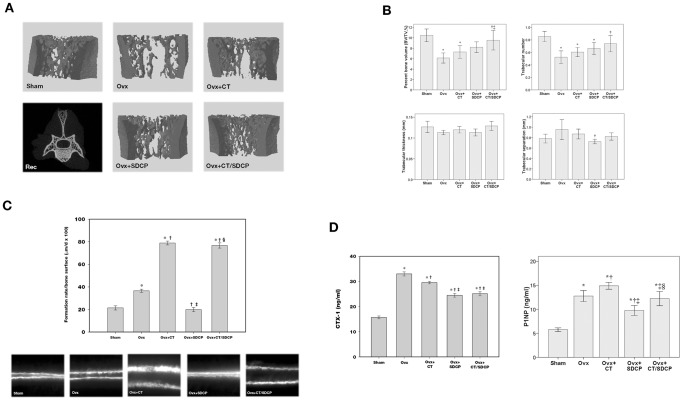
Calcitonin increases therapeutic efficacy of SDCP to treat osteoporotic rats. (A) Micro-computed tomography analysis of 5^th^ lumbar vertebrate in ovariectomized (OVX) rats treated with CT (5 IU/kg/day), SDCP (1 mg/kg/day), or both. Figures are representative 3D reconstruction images from each treatment groups except lower left panel which is 2D reconstruction image shows the contouring method used to delineate the trabecular bone region. (B) Quantitative results of the experiment shown in panel A. (C) Calcein double-labeling in OVX rats. Representative fluorescent micrographs show that the distance between the two labeled mineralization fronts. The quantification of the bone formation rate per bone surface is shown in the right panel. (D) Serum bone resorption marker (CTX-1) and bone formation marker (P1NP) were determined by ELISA. P<0.05 compared to *sham group, †Ovx group, ‡Ovx+CT group, and §Ovx+SDCP group after Bonferroni adjustment, mean ± SD, n = 6 in each group.

The influence of calcitonin-SDCP cotreatment on bone deposition was determined in an in vivo model of osteoporosis (Fig. 7). Ovariectomy or sham operation was performed in 3-month-old female Sprague Dawley rats. After four weeks, ovariectomized rats received normal saline, calcitonin, SDCP, or calcitonin plus SDCP treatment for four additional weeks after which micro-computed tomography analysis of the fifth lumbar vertebrates and histomorphometric analysis of the tibia were undertaken on the five-month-old rats. Although the 2D images also included cortical bone, regions of interest containing trabecular bone were selected for subsequent quantification. Significant increases in bone loss were observed in ovariectomized rats as compared to those of the sham controls (Fig. 7A and B). As compared to untreated ovariectomized rats, calcitonin treatment significantly increased percent bone volume and trabecular number. Compared to calcitonin treatment, SDCP induced further but not significant changes in these bone parameters; however, cotreatment with calcitonin and SDCP significantly increased percent bone volume and trabecular number in ovariectomized rats (Fig. 7A and 7B). No additional benefits in trabecular separation and thickness were observed upon cotreatment with calcitonin and SDCP. As shown in Figure 7C, a significant increase in bone formation was observed in ovariectomized rats, which was further increased upon calcitonin treatment. SDCP treatment in ovariectomized rats decreased bone formation, which was reversed by cotreatment with calcitonin (Fig. 7C).

Analysis of bone resorption and formation markers was next carried out after various treatments with calcitonin and SDCP (Fig. 7D). Analysis of a serum bone resorption marker, CTX-I, revealed increased bone resorption in ovariectomized rats as compared to those receiving the sham operation (Fig. 7D). Significant decreases in CTX-I were found in the calcitonin treatment group as compared to the untreated group. In both SDCP treatment and cotreatment groups, a further reduction in CTX-I was observed. Increases in the serum bone formation marker, P1NP, were observed in ovariectomized rats as compared to those receiving the sham operation (Fig. 7D). Further increases in P1NP levels were observed in ovariectomized rats receiving calcitonin. Although decreased P1NP levels were observed in the SDCP treatment group as compared to the calcitonin and vehicle groups, a significant increase was found in ovariectomized rats receiving the combination treatment of calcitonin and SDCP.

## Discussion

[LOSSEST]Because calcitonin may prolong osteoclast survival through inhibition of apoptosis, this study aimed to analyze its influence on osteoclast apoptosis induced by a bisphosphate analog, SDCP. Cotreatment of calcitonin and SDCP was chosen because the in vivo therapeutic effects of SDCP were comparable to those observed for alendronate, a conventional bisphosphonate [20]. In addition, the efficacy of cotreatment with these agents was explored in ovariectomized rats. In primary osteoclast cultures, calcitonin inhibited SDCP-induced apoptosis, resulting in increased osteoclast number and size. However, the SDCP-induced reduction in bone resorption was not affected by calcitonin. Furthermore, an additive effect of calcitonin and SDCP on increased bone formation, increasing bone volume, was observed in ovariectomized rats.

In contrast to most other antiresorptive agents, calcitonin treatment inhibits the function but not the survival of osteoclasts [22]. Indeed, calcitonin may play an important role in osteoclast survival by preventing apoptosis [10], [13]–[15]. To decrease bone resorption by osteoclasts, calcitonin induces cytoskeletal changes [23], causing cell detachment [11], decreasing mobility [24], and interaction with integrin-related signaling [10]. As in osteoclasts cultured on glass or bone [13], the present study showed that calcitonin promoted osteoclast survival in cells treated with SDCP by reducing apoptosis as observed by reduced chromatin condensation and DNA degradation, annexin-V staining, and caspase-3 cleavage. However, little activation of caspase 8 was observed in osteoclasts treat with calcitonin or SDCP, indicating that calcitonin and SDCP may not function through the extrinsic apoptotic pathway. This notion is corroborated by the effect of these agents on Bcl-2 family proteins. Specifically, calcitonin alone and combined with SDCP increased Bcl-2 expression in osteoclasts. In addition, SDCP reduced Mcl-1 expression, which was partially reversed with calcitonin cotreatment. These results indicate that calcitonin inhibits osteoclast apoptosis at least in part by increasing Bcl-2 expression, whereas SDCP treatment induces apoptosis by decreasing Mcl-1 expression. Consistent with these results, Sutherland et al. [25] reported that alendronate induced osteoclast apoptosis by decreasing Mcl-1 levels, which is prevented by co-treatment with RANKL. Further studies will be undertaken to elucidate the mechanism by which SDCP reduces Mcl-1 expression.

The antiapoptotic proteins Mcl-1, Bcl-2, and Bcl-xL differentially inhibit activator BH3-only proteins Bim, Bid, and Puma, which can directly activate Bak and Bax [26]. In the present study, the BH3-mimetic molecule, HA14-1, blocked the calcitonin-mediated reduction in caspase-3 and -9 activation. Thus, upregulation of Bcl-2 by calcitonin is likely the mechanism by which calcitonin prevents SDCP-induced osteoclast apoptosis.

Calcitonin receptor couples to multiple G proteins and activates multiple signaling pathways [27]. In osteoclasts, calcitonin activates Erk1/2 [15], mediating osteoclast survival [28]. Erk promotes cell survival by direct phosphorylation and inhibition of caspase-9 during development and tissue homeostasis [29]. In the present study, calcitonin-induced Bcl-2 expression, and inhibition of caspase-3 and -9 activation were sensitive to the Erk1/2 inhibitor, PD98059, confirming that Erk1/2 is involved in the antiapoptotic signaling induced by calcitonin in osteoclasts.

To achieve more powerful therapeutic effects, a combination of multiple anti-resorption agents might be needed for osteoporosis patients. Ogawa et al. [30] showed an additive effect on trabecular architecture and bone strength in ovariectomized rats after cotreatment with calcitonin and alendronate. In addition, a combined therapy of calcitonin and alendronate in patients with rheumatoid arthritis for 12 months had additive effects, significantly increasing lumbar and hip bone mineral density [31]. However, Iwamoto et al. [32] reported no significant changes in lumbar bone mineral density in postmenopausal osteoporotic women treated with alendronate and calcitonin compared to those receiving alendronate alone. This may be due to the short treatment period (6 months) [32]. In the present study, both calcitonin and SDCP increased the percent bone volume and trabecular number and thickness, and decreased trabecular separation in ovariectomized rats although not all these parameters reached significance. Calcitonin-SDCP cotreatment further increased percent bone volume, illustrating an additive effect by the combined treatment for osteoporosis. Further studies are necessary to determine whether combined therapy of pyrophosphate analog and calcitonin may represent a new strategy for osteoporosis treatment.

A balance between osteoblast and osteoclast activity is required for normal bone formation and maintenance. A coupling mechanism has been described in which resorption products and osteoclast-derived factors stimulate bone formation by osteoblast lineage cells [33]. Similarly, osteoblast lineage cells regulate osteoclast formation and activity [33]. Therefore, because of the coupled nature of remodeling, most of the anti-resorptive agents available also reduce bone formation directly or indirectly, limiting their effect on bone mass. Drugs that uncouple bone resorption from bone formation (e.g. inhibitors of chloride channels, cathepsin K, vacuolar H+ATPase and Src) may have a greater effect in terms of increasing bone mass. In the present study, combined therapy of calcitonin and SDCP had less of an effect on inhibiting bone resorption and induced more bone formation as compared to SDCP treatment alone as observed by pit formation assay, CTX-1 and P1NP expression, and calcein double labeling. Consistent with these results, calcitonin increased bone formation in ovariectomized rats [34], [35] and anabolic effect was observed in glucocorticoid-induced osteopenia [36]. Though results from calcitonin and calcitonin receptor-null mice suggest that it is a physiologic inhibitor of bone formation, the mechanism of how calcitonin regulates bone formation remains unknown [37]. The results from the present study suggest that calcitonin-induced inhibition of osteoclast apoptosis may be an important factor in this process.

There are some study limitations that warrant further discussion. For example, this study analyzes the effect of combination therapy using the bisphosphonate analog, SDCP, without comparing them with a conventional bisphosphonate, such as alendronate. In addition, the effects of SDCP alone or with calcitonin on osteoblasts were not assessed in the present study. However, Sun et al. [38] reported that SDCP inhibited osteoblast proliferation, which was mediated by the promotion of osteoblast differentiation and the increased synthesis of prostaglandin E2. Also, the effect of calcitonin and SDCP on apoptosis was only addressed in vitro without further studies seeding the osteoclasts on bone. Because this study will permit a correlation of anti-resorptive effects versus apoptosis, it will be carried out in future studies. Furthermore, 3-month old rats were used for the *in vivo* study. Because their skeletons are immature, the effect of bone growth cannot be separated from the effect of calcitonin or SDCP treatment to the rat skeleton. However, by the end of the study, the rats were five months of age. In conclusion, this study confirmed that calcitonin inhibits osteoclast apoptosis; it inhibited SDCP-induced apoptosis in primary osteoclast cultures. In addition, calcitonin-SDCP combination therapy inhibited bone resorption to a lesser extent and induced more bone formation as compared to SDCP alone. The benefits of combination therapy for osteoporosis warrant further investigation as a possible treatment for postmenopausal osteoporosis.

## Materials and Methods

### Reagents

Salmon calcitonin was purchased from Sigma-Aldrich (St. Louis, MO, USA). SDCP was purchased from Purzer Pharmaceutical Co., Taipei, Taiwan. TGF-βI was obtained from Peprotech (Rockey Hill, NJ, USA). HA14-1, a Bcl-2 inhibitor, was purchased from Enzo Life Science (Plymouth Meeting, PA, USA). PD98059, a MEK1 inhibitor, was purchased from Cell Signaling Technology (Danvers, MA, USA). Fas ligand (FasL) was purchased from R&D System (Minneapolis, MN, USA).

### Isolation and Treatment of Rabbit Osteoclasts

New Zealand white rabbits approximately 7-day-old (90–120 g) were used as the source of bone cells for purification of osteoclasts as previously described [11]. For subsequent imaging analyses, unpurified cultures were employed; however, purified cultures (>90% purity) were used for the molecular analysis. Purification of the cultures was performed using 0.02% EDTA and 0.001% pronase E for 5 min at 37°C. The experiment was performed with the approval of the Laboratory Animal Center of the National Defense Medical Center in Taipei, Taiwan. Purified osteoclasts were cultured in alpha-minimum essential medium (α-MEM, Sigma-Aldrich), pH 6.9, supplemented with 26 mM sodium bicarbonate, 10 mM HEPES, 1% penicillin/streptomycin, and 5% fetal bovine serum (FBS, HyClone, Logan, Utah, USA). Unless otherwise indicated, osteoclasts were treated with CT (10 nM), SDCP (10 µM), or both for 18 h for short-term changes including analysis of signaling molecules to 48 h for TUNEL and TRAP analysis.

### Confocal Microscopic Analysis of Osteoclast Apoptosis

1.5×10^4^ osteoclasts were cultured on 22×22 mm glass coverslips for 18 h after which they were treated with CT (10 nM), SDCP (10 µM) or both for 48 h. After the cells were washed with phosphate buffered saline (PBS), they were fixed in 4% paraformaldehyde for 10 min and permeabilized with 0.1% Triton X-100 in PBS for 5 min. The cells were subsequently incubated in 1% bovine serum albumin (BSA) in PBS for 1 h. Apoptosis was determined using an In Situ Cell Death Kit (Roche Applied Science, Mannheim, Germany) based on the TdT-mediated fluorescein dUTP nick-end labeling (TUNEL) method, according to the recommendations of the manufacturer. Negative controls included omission of TdT, and positive controls included DNase I treatments. Nuclei were counterstained using TOTO-3 diluted 1∶5000 (Molecular Probes, Inc., Eugene, OR, USA). For each treatment, at least 500 osteoclasts in three glass coverslips were counted. Measurement of apoptosis was calculated as a percentage of apoptotic nuclei versus total nuclei [39].

Apoptosis of cells was also determined by analysis of Annexin Vas well as cleaved caspase staining. 1.5×10^4^ osteoclasts were cultured on 22×22 mm glass coverslips for 18 h after which they were treated with CT (10 nM), SDCP (10 µM) or both for 18 h. For Annexin V staining, an Annexin V-FITC Apoptosis Detection kit (BioVision, Mountain View, CA, USA) was used, according to the recommendation of the manufacturer. Propidium iodide (50 µg/ml) was added to the binding buffer to detect necrosis. For analysis of cleaved caspase-3, cells were incubated with anticleaved caspase-3 polyclonal antibody (Cell Signaling Technology) in 1% BSA PBS at 4°C for 16 h, and then with Cy-3-conjugated anti-rabbit IgG antibody (Jackson Immunoresearch Laboratories, West Grove, PA, USA) in PBS for 1 h. Microscopy was performed using a confocal microscope equipped with difference interference contrast light path (LSM 510, Zeiss, Göttingen, Germany). Annexin V- and active caspase-3–positive osteoclasts were counted manually as stated above, and the apoptosis rate was statistically analyzed. Multi-nucleated cells were considered osteoclasts if they had more than three nuclei.

### Tartrate-resistant Acid Phosphatase (TRAP) Staining

1.5×10^4^ osteoclasts were cultured on 22×22 mm glass coverslips for 18 h after which they were treated with CT (10 nM), SDCP (10 µM) or both for 72 h. They were then stained for TRAP using a kit that uses 50 mM tartrate and following the manufacturer’s instructions (Sigma-Aldrich). For each treatment, at least 500 osteoclasts in three glass coverslips were counted. Osteoclasts were defined as cells with more than three nuclei. The number of TRAP^+^ cells per coverslip was determined by light microscope (Axio Imager A2, Zeiss).

### Pit Formation Assay

In the Pit formation assay, 1×10^3^ osteoclasts were cultured on dentine discs (Immunodiagnostic Systems Inc, Fountain Hills, AR) in a 96-well plate for 18 h after which they were treated with CT (10 nM), SDCP (10 µM),or both for 72 h. For most groups, there were 4 dentine discs per group. To measure the areas containing resorption lacunae, cells were removed, and the dentine discs were incubated in 0.25 M ammonium hydroxide, washed with distilled water, and then stained with 0.5% (wt/vol) toluidine blue. The images of the resorbed areas were measured using a reflective optical microscope (LSM 510, Zeiss), and the results were expressed as the number of resorption pits and total area resorbed per dentine disc.

### Western Blot Analysis

Purified osteoclasts (1.5±0.5×10^5^/10 cm dish) were cultured in α-MEM for 18 h after which they were treated with CT (10 nM), SDCP (10 µM), or both for 18 h. They were then washed twice with PBS, and lysed with cold lysis buffer (150 mM NaCl, 50 mM Tris, pH 7.5, 0.25% sodium deoxycholate, 0.1% Nonidet P-40, 1 mM sodium orthovanadate, 1 mM sodium fluoride, 1 mM phenylmethylsulfonyl fluoride, 10 µg/mL aprotinin, and 10 µg/mL leupeptin). The cell lysate was obtained by centrifugation at 16,000×g at 4°C for 30 min. The protein concentration was measured with a Bicinconinic acid kit (Pierce, Rockford, IL, USA), and 30 µg of total protein was separated on a 12% SDS-polyacrylamide gel. After the proteins were transferred to nitrocellulose membranes (Whatman, Dassel, Germany), the membranes were blocked with 5% skim milk in TBS-T (20 mM Tris, pH 7.6, 137 mM NaCl, and 0.1% Tween-20) and incubated with antibodies specific for cleaved caspase-3, 8, and 9 (Cell Signaling Technology) or anti-Bcl-2 and anti-Mcl-1(Santa Cruz, CA, USA). Proteins were visualized using the appropriate secondary antibody conjugated to horseradish peroxidase (HRP; Santa Cruz), followed by the application of ECL reagents (Amersham, Buckinghamshire, UK). The bands were quantified by densitometry (ProXPRESS Proteomic Imaging System, Perkin Elmer, Melbourne, VIC, Australia), and normalized to the loading control, actin. The influence of various treatments was expressed as fold-increase or decrease relative to the control lane. Each analysis was carried out in at least three independent experiments.

### In vivo Osteoporosis Model

Fifty 3-month-old female Sprague–Dawley rats were purchased from the Laboratory Animal Center, National Defense Medical Center (Taipei, Taiwan) and acclimated under standard laboratory conditions at 22±2°C and 50±10% humidity. Food and water were available ad libitum during the acclimatization period. A sham-operation (n = 10) or ovariectomy (OVX, n = 40) was performed. Four weeks after the surgery, OVX rats were divided into four groups: vehicle, calcitonin (50 IU/ml, subcutaneous injection of 5 IU/kg/day), SDCP (2.5 mg/ml, oral administration of 1 mg/kg/day), and calcitonin+SDCP (n = 10 for each group). Rats were treated five times per week for four weeks. The sham and OVX vehicle groups received the vehicle, an isotonic sodium chloride solution, orally. SDCP was administered orally by catheter at 1.0 mg/kg/day five times per week as previously described [20]. Salmon calcitonin (Miacalcic by Novartis-Pharma) was administered by subcutaneously injection at 5 IU/kg/day five times per week. The vehicle and drug were given on the same days, and the treatment period lasted 4 weeks. The animal study was carried out in accordance with ethical guidelines for animal care, and the experimental protocols were approved by the animal care committee of National Defense Medical Center.

Whole blood samples were obtained with plastic syringes via intracardiac puncture immediately following sacrifice at five months of age (8 weeks after surgery). The blood samples were allowed to clot at room temperature after which the serum was separated by centrifugation, divided into 500-µL aliquots, and stored at −80°C until further analysis. The lumbar vertebrae were also removed and stored at −80°C for subsequent assessment of trabecular microstructure.

### Micro-computed Tomography

Microarchitecture of the fifth lumbar trabecular bone was investigated using a microcomputed tomography (Skyscan 1174; Skyscan, Aartselaar, Belgium) as previously described [40]. The X-ray source was set at 50 kV, with a pixel size at 11 µm. Four hundred projections were acquired over an angular range of 180° (angular step of 0.45°). The image slices were reconstructed using the cone-beam reconstruction software version 2.6 based on the Feldkamp algorithm (Skyscan). The registered data sets were segmented into binary images. Simple global thresholding methods were used due to the low noise and relatively good resolution of the data sets. The trabecular bone was extracted by drawing ellipsoid contours with the CT analyzer software (Skyscan). Trabecular bone volume (BV/TV; percentage), trabecular number, and trabecular separation were calculated by the mean intercept length method. Trabecular thickness was calculated according to the method of Hildebrand and Ruegsegger [41].

### Histomorphometric Analysis

Eight weeks after ovariectomy, the rats received an intraperitoneal injection of calcein (30 mg/kg) and another injection 8 days later. Two days later they were sacrificed [42]. The proximal tibia were dehydrated in graded ethanol, defatted in acetone, and embedded undecalcified in London resin (London Resin Co., London, United Kingdom) after staining with Villanueva bone stain (Polyscience Ltd, Warrington, PA, USA). Frontal sections of the tibiae (7 µm thick) were prepared. Measurements were performed on the entire marrow region within the cortical shell of the proximal tibia metaphysis from 1–4 mm distal to the growth plate-metaphyseal junction using an Image Analysis System (Osteomeasure, Inc., Atlanta, GA, USA). Bone area, perimeter, single- and double-labeling surface were measured, and trabecular number, thickness, and separation as well as the bone formation rate/bone surface (BFR/BS) were calculated.

### Biochemical Analyses

Serum type 1 carboxyterminal collagen fragments (CTX-1) were measured using the RatLaps enzyme immunoassay (EIA; Immunodiagnostic Systems, UK), and the amino-terminal propeptide of type 1 procollagen (P1NP) was measured using the Rat PINP EIA (Immunodiagnostic Systems), according to the manufacturer’s instructions.

### Statistical Analysis

Means and standard deviations (SDs) were calculated for each group. Comparisons were performed using ANOVA with post-hoc comparison adjusted by the Bonferroni method. Data were analyzed with SAS 9.0 (SAS Institute Inc., Cary, NC), and a *P* value <0.05 was considered statistically significant.

## References

[1] Rogers MJ, Frith JC, Luckman SP, Coxon FP, Benford HL (1999). Molecular mechanisms of action of bisphosphonates.. Bone.

[2] Drake MT, Clarke BL, Khosla S (2008). Bisphosphonates: mechanism of action and role in clincal practice.. Mayo Clin Proc.

[3] Rogers MJ, Crockett JC, Coxon FP, Mönkkönen J (2011). Biochemical and molecular mechanisms of action of bisphosphonates.. Bone.

[4] Luckman SP, Hughes DE, Coxon FP, Graham R, Russell G (1998). Nitrogen-containing bisphosphonates inhibit the mevalonate pathway and prevent post-translational prenylation of GTP-binding proteins, including Ras.. J Bone Miner Res.

[5] Karsdal MA, Henriksen K, Arnold M, Christiansen C (2008). Calcitonin: a drug of the past or for the future? Physiologic inhibition of bone resorption while sustaining osteoclast numbers improves bone quality.. BioDrugs.

[6] de Paula FJ, Rosen CJ (2010). Back to the future: revisiting parathyroid hormone and calcitonin control of bone remodeling.. Horm Metab Res.

[7] Gallagher JC, Sai AJ (2010). Molecular biology of bone remodeling: implications for new therapeutic targets for osteoporosis.. Maturitas.

[8] Woodrow JP, Sharpe CJ, Fudge NJ, Hoff AO, Gagel RF (2006). Calcitonin plays a critical role in regulating skeletal mineral metabolism during lactation.. Endocrinology.

[9] Davey RA, Turner AG, McManus JF, Chiu WS, Tjahyono F (2008). Calcitonin receptor plays a physiological role to protect against hypercalcemia in mice.. J Bone Miner Res.

[10] Marzia M, Chiusaroli R, Neff L, Kim NY, Chishti AH (2006). Calpain is required for normal osteoclast function and is down-regulated by calcitonin.. J Biol Chem.

[11] Shyu JF, Shih C, Tseng CY, Lin CH, Sun DT (2007). Calcitonin induces podosome disassembly and detachment of osteoclasts by modulating Pyk2 and Src activities.. Bone.

[12] Stern PH (2007). Antiresorptive agents and osteoclast apoptosis.. J Cell Biochem.

[13] Selander KS, Härkönen PL, Valve E, Mönkkönen J, Hannuniemi R (1996). Calcitonin promotes osteoclast survival in vitro.. Mol Cell Endocrinol.

[14] Kanaoka K, Kobayashi Y, Hashimoto F, Nakashima T, Shibata M (2000). A common downstream signaling activity of osteoclast survival factors that prevent nitric oxide-promoted osteoclast apoptosis.. Endocrinology.

[15] Miyazaki T, Neff L, Tanaka S, Horne WC, Baron R (2003). Regulation of cytochrome c oxidase activity by c-Src in osteoclasts.. J Cell Biol.

[16] Hoff AO, Catala-Lehnen P, Thomas PM, Priemel M, Rueger JM (2002). Increased bone mass is an unexpected phenotype associated with deletion of the calcitonin gene.. J Clin Invest.

[17] Huebner AK, Schinke T, Priemel M, Schilling S, Schilling AF (2006). Calcitonin deficiency in mice progressively results in high bone turnover.. J Bone Miner Res 2006.

[18] Lin FH, Lin CC, Lu CM, Liu HC, Sun JS (1995). Mechanical properties and histological evaluation of sintered beta-Ca2P2O7 with Na4P2O7.10H2O addition.. Biomaterials.

[19] Sun JS, Tsuang YH, Liao CJ, Liu HC, Hang YS (1997). The effects of calcium phosphate particles on the growth of osteoblasts.. J Biomed Mater Res.

[20] Sun JS, Huang YC, Tsuang YH, Chen LT, Lin FH (2002). Sintered dicalcium pyrophosphate increases bone mass in ovariectomized rats.. J Biomed Mater.

[21] Sun JS, Huang YC, Lin FH, Chen LT (2003). The effect of sintered dicalcium pyrophosphate on osteoclast metabolism: an ultrastructural study.. J Biomed Mater Res A.

[22] Karsdal MA, Henriksen K, Arnold M, Christiansen C (2008). Calcitonin- A drug of the Past or for the Future?. BioDrugs.

[23] Chambers TJ, Athanasou NA, Fuller K (1984). Effect of parathyroid hormone and calcitonin on the cytoplasmic spreading of isolated osteoclasts.. J Endocrinol.

[24] Zaidi M, Chambers TJ, Moonga BS, Oldoni T, Passarella E (1990). A new approach for calcitonin determination based on target cell responsiveness.. J Endocrinol Invest.

[25] Sutherland KA, Rogers HL, Tosh D, Rogers MJ (2009). RANKL increases the level of Mcl-1 in osteoclasts and reduces bisphosphonate-induced osteoclast apoptosis in vitro.. Arthritis Res Ther.

[26] Tanaka S, Wakeyama H, Akiyama T, Takahashi K, Amano H (2010). Regulation of osteoclast apoptosis by bcl-2 family protein bim and caspase-3.. Adv Exp Med Biol.

[27] Horne WC, Sanjay A, Baron R (2008). Regulating Bone Resorption: Targeting Integrins, Calcitonin Receptor, and Cathepsin K, in: J.P. Bilezikian, T.J. Martin (Eds.) Principles of Bone Biology 3rd ed., Academic Press, New York, NY, USA, 221–236..

[28] Miyazaki T, Katagiri H, Kanegae Y, Takayanagi H, Sawada Y (2000). Reciprocal role of ERK and NF-kappaB pathways in survival and activation of osteoclasts.. J Cell Biol.

[29] Allan LA, Morrice N, Brady S, Magee G, Pathak S (2003). Inhibition of caspase-9 through phosphorylation at Thr 125 by ERK MAPK.. Nat Cell Biol.

[30] Ogawa K, Hori M, Takao R, Sakurada T (2005). Effects of combined elcatonin and alendronate treatment on the architecture and strength of bone in ovariectomized rats.. J Bone Miner Metab.

[31] Ozoran K, Yildirim M, ÖNDER M, Sivas F, Inanir A (2007). The bone mineral density effects of calcitonin and alendronate combined therapy in patients with rheumatoid arthritis.. APLAR J Rheumatol.

[32] Iwamoto J, Uzawa M, Sato Y, Takeda T, Matsumoto H (2009). Effects of short-term combined treatment with alendronate and elcatonin on bone mineral density and bone turnover in postmenopausal women with osteoporosis.. Ther Clin Risk Manag.

[33] Martin T, Gooi JH, Sims NA (2009). Molecular mechanisms in coupling of bone formation to resorption.. Crit Rev Eukaryot Gene Expr.

[34] Mochizuki K, Inoue T (2000). Effect of salmon calcitonin on experimental osteoporosis induced by ovariectomy and low-calcium diet in the rat.. J Bone Miner Metab.

[35] Davey RA, Morris HA (2005). The effects of salmon calcitonin-induced hypocalcemia on bone metabolism in ovariectomized rats.. J Bone Miner Metab.

[36] Furuichi H, Fukuyama R, Izumo N, Fujita T, Kohno T (2000). Bone-anabolic effect of salmon calcitonin on glucocorticoid-induced osteopenia in rats.. Biol Pharm Bull.

[37] Huebner AK, Keller J, Catala-Lehnen P, Perkovic S, Streichert T (2008). The role of calcitonin and alpha-calcitonin gene-related peptide in bone formation.. Arch Biochem Biophys.

[38] Sun JS, Tsuang YH, Liao CJ, Liu HC, Hang YS (1999). The effect of sintered beta-dicalcium pyrophosphate particle size on newborn Wistar rat osteoblasts.. Artif Organs.

[39] Penolazzi L, Lampronti I, Borgatti M, Khan MT, Zennaro M (2008). Induction of apoptosis of human primary osteoclasts treated with extracts from the medicinal plant Emblica officinalis.. BMC Complement Altern Med.

[40] Bouxsein ML, Boyd SK, Christiansen BA, Guldberg RE, Jepsen KJ (2010). Guidelines for assessment of bone microstructure in rodents using micro-computed tomography.. J Bone Miner Res.

[41] Hildebrand T, Ruegsegger P (1997). Quantification of Bone Microarchitecture with the Structure Model Index.. Comput Methods Biomech Biomed Engin.

[42] Lin C, Moniz C, Chambers TJ, Chow JWM (1996). Colitis causes bone loss in rats through suppression of bone formation.. Gastroenterol.

